# High altitude renal syndrome: four elements or one source?

**DOI:** 10.1080/0886022X.2025.2482125

**Published:** 2025-04-03

**Authors:** Lei Zhang, Erchao Feng, Sezhen Baima, Cangjue Laba, Lazhen Suolang, Ji Cang, Qing De

**Affiliations:** ^a^ The First Affiliated Hospital of Anhui University of Chinese Medicine, Hefei, Anhui Province, China; ^b^ Center for High Altitude Disease Diagnosis and Treatment/Renal Department, Shannan People’s Hospital Shannan City Tibet Autonomous Region, China

**Keywords:** High altitude renal syndrome, high altitude polycythemia, hypoxia, pathogenesis, carbonic anhydrase inhibitors, altitude descent

## Abstract

High Altitude Renal Syndrome (HARS) is a clinical syndrome characterized by polycythemia, hyperuricemia, hypertension, and albuminuria at high altitudes. This review emphasizes hypoxia-induced High Altitude Polycythemia (HAPC) as its core driver. In 2014, the 6th Qinghai International Conference on Mountain Medicine and High Altitude Physiology established international diagnostic criteria for HAPC (hemoglobin ≥210g/L in males, ≥190g/L in females). HAPC triggers systemic hypoxia, leading to hyperuricemia via uric acid overproduction and reduced renal excretion, hypertension from hemodynamic changes, and glomerular injury through erythrocytosis. Pathological mechanisms involve HIF-2α-mediated glomerular hypertrophy and podocyte damage. Treatment strategies target HAPC and downstream effects. Carbonic anhydrase inhibitors (e.g., acetazolamide) reduce hematocrit and improve oxygen saturation, but newer agents like methazolamide and dichlorphenamide offer equivalent efficacy with fewer side effects (e.g., reduced central nervous system toxicity). For severe cases, descending to lower altitudes remains the gold standard. Additional interventions include calcium channel blockers (nifedipine), urate-lowering drugs, and experimental therapies such as HIF-2α inhibitors (PT2385) and endothelin receptor antagonists (matitan). This analysis underscores HAPC as the primary etiology of HARS, advocating revised diagnostic criteria and treatment prioritization.

## Introduction

High Altitude Renal Syndrome (HARS) was first proposed by Arestegui from Peru in 2011. Patients mainly present with a clinical syndrome characterized by erythrocytosis, hyperuricemia, hypertension, and albuminuria at high altitudes (above 2500 meters) [1]. Compared to the damage to cardiopulmonary function caused by high altitude, HARS is rarely recognized. Although there have been recent reports of related cases [2], the diagnosis rate of HARS remains low. This article provides a detailed explanation of the epidemiology, pathophysiological mechanisms, and treatment progress of HARS, in order to reexamine the diagnosis and treatment of this disease.

### High altitude polycythemia

High Altitude Polycythemia (HAPC) is a necessary condition for the diagnosis of HARS. HAPC is a manifestation of excessive compensatory proliferation of red blood cells caused by hypoxia at high altitudes, usually occurring in residents who have lived in areas above 2500 meters above sea level for a long time. The main characteristics are abnormally elevated red blood cell count and hemoglobin concentration [3]. In 2014, the 6th Qinghai International Conference on Mountain Medicine and High Altitude Physiology established the international diagnostic criteria for HAPC (referred to as the Qinghai Criteria), which are a hemoglobin concentration of ≥210 g/L for males and ≥190 g/L for females [4]. Whether newly arrived or long-term residents in high-altitude areas, the risk of HAPC is significantly increased compared to those in plain areas. The hemoglobin level in populations living in high-altitude areas of Saudi Arabia is approximately 169.8 ± 10.63 g/L, which is higher than the 144.6 ± 13.96 g/L in low-altitude areas [5]. In the Qinghai-Tibet Plateau region of China, the incidence of HAPC among newly arrived populations is 25.8% [6]. For long-term residents of high-altitude areas, HAPC is mainly related to genes associated with hypoxic adaptation and specific gene methylation. These regions primarily involve genes in the HIF pathway, such as EPAS1, EGLN1, and PPARA expression [7], and increased methylation levels of BMPR2 and TGF-β [8].

Polycythemia can directly cause kidney damage, clinically presenting as acute kidney injury, chronic kidney disease, and acute tubular necrosis; the degree of renal lesions is related to the severity of polycythemia [9]. In terms of pathological types, polycythemia can lead to IgA nephropathy [10], minimal change disease [11], and focal segmental glomerulosclerosis [12]. Pathological features of HAPC-related kidney damage include glomerular hypertrophy, thickening of the glomerular basement membrane, podocyte injury, and peripheral arterial/arteriolar lesions. The expression of HIF-2α is closely related to the onset of the disease, indicating that chronic hypoxia and secondary hemodynamic changes may be the main pathogenesis of the disease [13]. This mechanism has also been fully confirmed in animal experiments, where newborn dogs with acute polycythemia had a significant increase in blood viscosity, a 55% decrease in renal plasma flow, and a 53% decrease in glomerular filtration rate [14]. The clinical characteristics of HAPC-related kidney disease are manifested as moderate to severe proteinuria, hyperuricemia, and hypertension, with possible renal insufficiency in the late stage [13]. This is nearly consistent with the clinical manifestations of HARS, so HAPC may be the fundamental cause of HARS.

### High altitude hyperuricemia

Serum uric acid can directly and indirectly promote kidney damage at the cellular and tissue levels through various pathogenic mechanisms [15]. Hyperuricemia (HUA) is an important and potential risk factor for kidney injury [16]. Although HARS is complicated by HUA, the occurrence of HUA in high-altitude areas is closely related to HAPC. As the altitude increases, the risk of HUA in the population gradually increases [6]. Early clinical observations have confirmed that serum uric acid levels in residents of high-altitude areas are positively correlated with hematocrit [17]. Recent epidemiological observations conducted in high-altitude areas in China have once again confirmed this phenomenon [18]. In addition, the same phenomenon has been observed in studies of high-altitude populations in Peru, and this study believes that the increase in uric acid levels is due to increased uric acid production caused by systemic hypoxia and impaired renal excretory function [19]. Related experimental research has found that a hypoxic environment leads to uric acid accumulation in rats, increased expression of desmin protein in podocytes, and decreased activity of Na+-K+-ATPase [20]. The increase in uric acid may be related to increased expression of liver xanthine oxidase and adenosine deaminase, as well as decreased expression of renal uric acid excretion-related transporters, organic anion transporter 1 and organic cation transporter 1 [21]. Therefore, for HARS patients without a history of HUA, the occurrence and development of HUA are directly related to systemic hypoxia and HAPC.

### High altitude hypertension

Kidney damage caused by hypertension involves a variety of complex molecular mechanisms, including the renin-angiotensin-aldosterone system, oxidative stress, endothelial dysfunction, and genetic and epigenetic factors [22], while clinically it is mainly manifested as persistent proteinuria [23], which also indicates that patients may face some adverse prognosis [24]. Cardiovascular adaptation in high-altitude environments is mainly related to factors such as sympathetic nervous system activation, myocardial suppression effect, hypocapnia, reduced blood volume, increased blood viscosity, and pulmonary hypertension [25]. HARS patients have elevated blood pressure, but this is mainly due to hypoxia at high altitudes. The prevalence of hypertension in the general population increases with the increase in residential altitude [26]. Newcomers to the plateau mainly show a consistently high diastolic blood pressure [27], in addition to a significant increase in hemoglobin concentration [28]. A cross-sectional study conducted in Garzê Tibetan Autonomous Prefecture (average altitude 3500 meters) showed that for every 1 g/L increase in hemoglobin concentration, the risk of hypertension increased by 1.02 times. Moreover, compared to normal hemoglobin concentration, the risk of hypertension increased by 2.92 times for HAPC, especially when the hemoglobin concentration exceeded about 176 g/L, the risk of hypertension significantly increased [29]. In summary, the occurrence and development of hypertension in HARS patients are also closely related to hypoxia and HAPC.

### High altitude kidney injury

HARS kidney injury is mainly manifested by the occurrence and development of proteinuria. Healthy populations living at high altitudes for a long time have a higher risk of kidney disease than those near sea level, characterized by worse renal function and a higher incidence of proteinuria [30]. In healthy individuals, the high-altitude hypoxic environment can increase the urinary albumin excretion rate from 3.2 μg/min to 5.0 μg/min [31]. There are differences in the pathological spectrum of kidney biopsy patients between highland and plain areas, with minimal change disease being the most common in highland areas, while IgA nephropathy is predominant in plain areas [32]. IgA nephropathy patients in highland areas have higher levels of proteinuria and more severe hypoproteinemia than those in plain areas, with higher glomerular filtration rates and a higher incidence of nephrotic syndrome [33]. Exposure to high-altitude hypoxic environments results in an approximate 11% decrease in glomerular filtration rate compared to normal sea level, while hematocrit increases by about 44% [34]. Even without the complications of hypertension, diabetes, or chronic kidney disease, residents of high-altitude areas have poorer renal function, and hemoglobin concentration is negatively correlated with glomerular filtration rate [30]. The complex structure of the kidney makes it marginally hypoxic even at sea level, especially in the outer medulla region. Acute hypoxia leads to increased urinary protein excretion due to changes in capillary permeability and altered glomerular filtration function [35], in addition, this also participates in the increase in blood pressure under acute hypoxic conditions [36]. Hypoxia-inducible factor-1α can induce high expression of Kruppel-like factor 4 mediated podocyte damage under hypoxic conditions, which is also one of the mechanisms of proteinuria occurrence in high-altitude environments [37].

### Treatment of HARS

Due to the impact of high-altitude exposure on physiology and pharmacokinetics, these are factors that need to be considered during treatment. High altitude leads to blood concentration, increased hemoglobin and total protein concentrations, thereby increasing the protein binding rate of drugs. However, blood concentration reduces the distribution volume of drugs and increases the clearance rate of drugs. High altitude increases the pH value of urine, thereby affecting drug excretion [38]. In the use of antihypertensive drugs, the half-life of diltiazem is prolonged, the half-life and area under the curve of nitrendipine increase, while the plasma clearance rate decreases [39]. Selective β1 receptor blockers have less impact on oxygen saturation and exercise tolerance in a hypoxic environment compared to nonselective β receptor blockers [40]. The angiotensin II receptor antagonist telmisartan is effective below an altitude of 3400 meters but may lose its antihypertensive effect at an altitude of 5400 meters, possibly due to the inhibition of the renin-angiotensin system by the hypoxic environment [41]. The calcium channel blocker nifedipine can effectively reduce pulmonary artery pressure and prevent high-altitude pulmonary edema under acute hypoxic exposure and does not require dose adjustment in a high-altitude hypoxic environment [42]. In addition to conventional drugs that inhibit uric acid production or promote uric acid excretion for hyperuricemia, oral vitamin C supplements (500 mg per day) can prevent hyperuricemia in high-altitude areas [43]. Although HARS is a clinical syndrome characterized by erythrocytosis, hyperuricemia, hypertension, and albuminuria, hypoxia and hypoxia-induced HAPC are the main factors mediating the above manifestations in patients. Relevant studies have shown that the blood uric acid levels and incidence of proteinuria in the HAPC population are significantly higher than those in other groups, and the degree of proteinuria is positively correlated with hematocrit and blood uric acid levels [19]. Therefore, active intervention and treatment of hypoxia and hypoxia-induced HAPC are important targets for controlling disease progression.

Carbonic anhydrase inhibitors have been proven to be useful for the prevention and treatment of high-altitude diseases. Currently, acetazolamide is widely reported. Both 250 mg/day and 500 mg/day can reduce hematocrit, increase nocturnal arterial oxygen saturation, and decrease nocturnal heart rate [44]. At an altitude of 5100 meters, acetazolamide 250 mg/day can significantly reduce hematocrit by 5.2% and hemoglobin concentration by 2.7% [45]. In the treatment of focal segmental glomerulosclerosis caused by HARS, patients received three sessions of 500 mL phlebotomy and used drugs including enalapril, spironolactone, atorvastatin, and aspirin. No significant reduction in urinary protein was observed. When the patient refused to use corticosteroids, acetazolamide 250 mg bid was administered. After 4 months, the hemoglobin concentration decreased from 23.2 g/dl to 19 g/dl, and the 24-h protein excretion decreased from 3,956 mg/d to 2,003 mg/d [12]. However, common side effects of acetazolamide include headache, decreased intraocular pressure, and cognitive decline. These side effects are similar to the symptoms of high-altitude sickness, which may lead to confusion in diagnosis and underestimation of drug efficacy. Therefore, methazolamide and dichlorphenamide have become effective alternative treatments. Compared to acetazolamide, methazolamide has several advantages, including higher liposolubility, less plasma protein binding, and renal excretion, with fewer side effects. Its efficacy in preventing and treating high-altitude diseases is similar to acetazolamide, and it performs better in reducing fatigue side effects [46]. Case reports from Peru also confirm this result. A 71-year-old female patient with chronic mountain sickness had a daytime resting oxygen saturation of 80%. Her symptoms improved after using oxygen (2 liters/minute). The patient began taking acetazolamide 250 mg twice daily, and three days later, her daytime oxygen saturation increased to 90%. Later, due to taste abnormalities caused by acetazolamide, she switched to methazolamide 50 mg twice daily, and her symptoms disappeared, with oxygen saturation maintained above 90% [47]. Compared to acetazolamide, dichlorphenamide has fewer side effects due to its lower lipophilicity and central nervous system penetration, especially in terms of the central nervous system. Clinical observations also confirm that dichlorphenamide 200 mg/day has fewer side effects compared to acetazolamide 500 mg/day [48].

In addition, some experimental treatments have shown effectiveness and may become new treatment methods in the future. PT2385, as a selective HIF-2α inhibitor, can be used for the treatment of high-altitude polycythemia model rats. Studies have found that after PT2385 intervention, the content of red blood cells and hemoglobin in rats significantly decreased, and angiogenesis, lipid peroxidation, and inflammatory responses were reduced [49]. Matitan, an endothelin receptor antagonist mainly used for the treatment of pulmonary arterial hypertension, has been found in experimental studies to alleviate chronic high-altitude sickness in rats by regulating arginine and purine metabolism [50]. Arginine can alleviate the symptoms of high-altitude sickness in rodents exposed to hypobaric hypoxia by regulating miR-144-5p to reduce erythropoietin/erythropoietin receptor [51]. Compared to drug intervention, descending to a lower altitude is the most effective treatment, which can reduce hemoglobin by 16%, while nocturnal oxygen supplementation and daily acetazolamide can only reduce hemoglobin by 6% [52]. Finally, active prevention is also an effective method. Firstly, ascending no more than 500 m per day and staying at the same altitude every 3–4 days to allow the body time to adapt to the high-altitude environment. Acetazolamide is used for prevention, but for severe cases, dexamethasone is recommended, and immediate descent to a lower altitude area is advised [53].

## Conclusions

HARS is a common clinical syndrome in high-altitude areas, characterized by erythrocytosis, hyperuricemia, hypertension, and albuminuria. Hypoxia and hypoxia-induced HAPC are the core factors in the occurrence and development of HARS. From current research, HAPC can explain the occurrence of hyperuricemia, hypertension, and albuminuria questioning the rationale for generating a distinct concept of HARS. Alternatively, it may be replaced by HAPC kidney damage. These concerns warrant further research and discussion. In the treatment of HARS, the impact of high-altitude exposure on pharmacokinetics must be fully considered. In addition to symptomatic treatment for blood pressure and uric acid, treatment should mainly target HAPC. Carbonic anhydrase inhibitors have clear therapeutic effects in the treatment of HAPC, achieving multiple benefits. Compared to traditional acetazolamide, methazolamide and dichlorphenamide have the advantages of equivalent efficacy and fewer side effects. However, for severe cases, descending to a lower altitude remains the best prevention and treatment method (Table 1).

**Table 1.** pathophysiology, clinical manifestations, and intervention strategies for four elements in HARS.

Elements	Pathophysiology	Clinical Manifestations	Intervention Strategies
High Altitude Polycythemia	The hypoxic environment at high altitude stimulates an increase in erythropoietin (EPO) production, leading to polycythemia and increased blood viscosity	An increase in hematocrit and hemoglobin concentration may lead to symptoms such as thrombosis, headache, and fatigue	Carbonic anhydrase inhibitors (such as acetazolamide, methazolamide, benzolamide) Chinese herbal medicines (such as Astragalus membranaceus, Duoxuekang Capsules) HIF-2α inhibitors (such as PT2385)
High Altitude Hyperuricemia	In a hypoxic environment, uric acid production increases while excretion decreases, leading to an elevation in blood uric acid levels	An elevated blood uric acid level may trigger complications such as gout and kidney stones	Drugs that inhibit uric acid production (such as allopurinol) Drugs that promote uric acid excretion (such as benzbromarone) Vitamin C (500mg per day)
High Altitude Hypertension	In a hypoxic environment, the sympathetic nerve is excited, causing blood vessels to constrict and blood pressure to rise	The elevation of blood pressure may cause symptoms such as headache, dizziness, and palpitations, and it also increases the risk of cardiovascular diseases	Calcium channel blockers (such as nifedipine) Non - selective beta - blockers Telmisartan combined with nifedipine Acetazolamide
High Altitude Kidney Injury	Polycythemia and hyperuricemia lead to a decrease in the glomerular filtration rate and an increase in proteinuria, which may trigger glomerulosclerosis	Proteinuria and a decrease in the glomerular filtration rate may lead to renal insufficiency	Carbonic anhydrase inhibitors (such as acetazolamide, methazolamide) Angiotensin - converting enzyme inhibitors (such as enalapril) Chinese herbal medicines (such as Longgui Yangxin Pills)

## Data Availability

No data were generated or analyzed in the current research.
